# Recent advances in the crosstalk between the brain-derived neurotrophic factor and glucocorticoids

**DOI:** 10.3389/fendo.2024.1362573

**Published:** 2024-04-05

**Authors:** Alexandros Tsimpolis, Konstantinos Kalafatakis, Ioannis Charalampopoulos

**Affiliations:** ^1^ Department of Pharmacology, Medical School, University of Crete, Heraklion, Greece; ^2^ Institute of Molecular Biology and Biotechnology, Foundation for Research and Technology Hellas (IMBB-FORTH), Heraklion, Greece; ^3^ Faculty of Medicine and Dentistry (Malta Campus), Queen Mary University of London, Victoria, Malta

**Keywords:** glucocorticoids, brain-derived neurotrophic factor, TrkB receptor, biorhythmicity, mood disorders, neurodegeneration, neuroinflammation

## Abstract

Brain-derived neurotrophic factor (BDNF), a key neurotrophin within the brain, by selectively activating the TrkB receptor, exerts multimodal effects on neurodevelopment, synaptic plasticity, cellular integrity and neural network dynamics. In parallel, glucocorticoids (GCs), vital steroid hormones, which are secreted by adrenal glands and rapidly diffused across the mammalian body (including the brain), activate two different groups of intracellular receptors, the mineralocorticoid and the glucocorticoid receptors, modulating a wide range of genomic, epigenomic and postgenomic events, also expressed in the neural tissue and implicated in neurodevelopment, synaptic plasticity, cellular homeostasis, cognitive and emotional processing. Recent research evidences indicate that these two major regulatory systems interact at various levels: they share common intracellular downstream pathways, GCs differentially regulate BDNF expression, under certain conditions BDNF antagonises the GC-induced effects on long-term potentiation, neuritic outgrowth and cellular death, while GCs regulate the intraneuronal transportation and the lysosomal degradation of BDNF. Currently, the BDNF-GC crosstalk features have been mainly studied in neurons, although initial findings show that this crosstalk could be equally important for other brain cell types, such as astrocytes. Elucidating the precise neurobiological significance of BDNF-GC interactions in a tempospatial manner, is crucial for understanding the subtleties of brain function and dysfunction, with implications for neurodegenerative and neuroinflammatory diseases, mood disorders and cognitive enhancement strategies.

## 1 Introduction

### 1.1 Glucocorticoids: chrono-modulators of behavior and neuroinflammatory responses

Glucocorticoids (GCs) have been traditionally considered as pivotal components of the stress response. They constitute the end-product of the hypothalamic-pituitary-adrenal (HPA) axis, a complex neuroendocrine system; they are synthesized by the adrenal cortex and secreted into the systemic circulation, reaching multiple targets within the mammalian body to exert their molecular effects The HPA axis comprises several key nodes that interact reciprocally, including cortical and subcortical brain regions such as the prefrontal cortex, amygdala, and hippocampus. These areas encode stressful cues and regulate the secretion of corticotropin-releasing hormone (CRH) by the hypothalamus, which in turn upregulates the secretion of corticotrophin (ACTH) by the anterior pituitary. Subsequently, ACTH stimulates the biosynthesis and secretion of GCs by the adrenal cortex. GCs feed back to the anterior pituitary and hypothalamus to inhibit the secretion of CRH and ACTH, creating a negative auto-feedback loop (1). High GC levels during a stress response facilitate the proper functioning of cells and tissues within a framework of increased metabolic and cognitive demands, in various cases, also requiring the mobilization of the immune system. Of equal importance, the reduction of GC levels at the post-stress period is crucial for facilitating the reestablishment of homeostasis and the successful, long-term management of the allostatic load (i.e., effective neurobehavioral, immunological and metabolic adaptation). Chronic stress may dysregulate the HPA axis, thus disrupting the fine temporal balance between appropriately high and appropriately low GC levels. This loss of synchronicity between when GC levels should be high/low (based on the bodily demands) and when they actually are, could have a role in the development of stress-related disorders, including post-traumatic stress disorder and Major Depressive Disorder (MDD) (2, 3).

But in the realms of neurobiology and neuroendocrinology, a nuanced understanding of the role of GCs in the brain physiology reveals a complex interplay that goes far beyond their well-characterized role in the stress response. GCs, mainly cortisol in humans and corticosterone in rodents, are steroid hormones that orchestrate a wide array of processes; from interactions at the genomic and molecular level, all the way to the regulation of behavioral responses (4, 5). Within the complexity of neural circuits and cellular interactions, these hormones wield a significant influence.

One of the key aspects that merits attention, is the circadian rhythmicity of GC secretion. Under basal conditions, GCs follow a dynamic circadian pattern with high GC levels right before and during the active phase and low levels at the inactive phase of the day (6). The brain’s circadian clock, centered in the suprachiasmatic nucleus (SCN) of the hypothalamus, orchestrates the diurnal release of GCs (7). This circadian variation in GC levels is vital for optimizing various neural functions, including memory consolidation, mood regulation, and cognitive performance (8–10). Dysregulation of this rhythm, mainly due to chronic stress, has been linked to cognitive impairments and mood disorders (11).

Going deeper into the complex GC biorhythmicity, recent sources of evidence illustrate that -under baseline conditions- GCs are secreted in a pulsatile manner, every hour or few hours (depending on the mammalian species), as a result of the positive feedforward, negative feedback interplay between the anterior pituitary and adrenal glands, which is not dependent on the hypothalamic input. This GC pulsatility also seems to have a neurobiological significance; absence of pulsatility (or changes in the characteristics of pulsatility, like the frequency or duration of pulses) impact for example the temporal pattern of the expression of GC-sensitive genes and synaptic plasticity in rodents (11), as well as the neural processing, cognitive and behavioral responses in man (11).

To further underscore their complex role in the central nervous system (CNS), GCs have also been implicated in the mediation of both anti-inflammatory and pro-inflammatory effects. Generally, GCs are well-known for their potent anti-inflammatory properties, a facet of their role that is highly exploited pharmaceutically, especially for the management of autoimmune disorders (12). In the context of the nervous system, acting mainly via the glucocorticoid (GR) and mineralocorticoid receptor (MR), GCs demonstrate this opposing effect on the progression of neuroinflammation, in a distinct temporal manner. More specifically, when GC administration is acute, short-termed and in high doses, the development of an anti-inflammatory environment is favored, mainly characterized by reduced microgliosis and astrogliosis, repression of pro-inflammatory cytokine expression (including TNF-α, IL-1α and IL-1β) and neuroprotection (13–15). On the other hand, chronic GC administration or hypersecretion, induces microgliosis, primes microglial activation and promotes pro-inflammatory cytokine overexpression and hippocampal accumulation (16–18).

Interestingly, this temporal distinction in duration and subsequent downstream regulation of pro- or anti-inflammatory action by GCs, perfectly correlates with the “double-edged sword” nature of neuroinflammation itself within the CNS (19); while it plays a protective role in response to injury or infection, chronic or excessive inflammation can lead to neuronal damage and contribute to neurodegenerative disorders.

### 1.2 Brain-derived neurotrophic factor: modulator of mood and neuroinflammatory responses

Brain-derived neurotrophic factor (BDNF) is a potent neuroprotective factor both during nervous system development and in adulthood, promoting neuronal survival, growth and synaptic plasticity, thus playing a crucial role in shaping the brain’s structure and function throughout life. In neurodegenerative conditions, where neurons are progressively lost, deficits in BDNF expression levels and signaling are often observed (20, 21). This deficiency can exacerbate the neurodegenerative process, compromising the brain’s ability to repair and maintain neural circuits. In Alzheimer’s Disease (AD), for instance, reduced BDNF levels are associated with the accumulation of beta-amyloid plaques and tau tangles (22, 23), while in Parkinson’s disease, BDNF stimulation can promote the dopaminergic neuron survival and differentiation in the substantia nigra (24).

BDNF, besides influencing neuronal growth and survival, also exhibits profound effects on mood regulation and emotional well-being (25). Stress is a well-known trigger for regulating BDNF levels in the brain, with the latter varying depending on the duration and the type of the stressor as well as the brain region. Chronic stress can lead to epigenetic alterations of the *Bdnf* gene, actively promoting its methylation and suppressing its expression, especially in the prefrontal cortex and the hippocampus (26). Furthermore, prolonged exposure to stress in adolescent rats induced an anxiety-like behavior that was directly correlated to their reduced BDNF protein levels (27). However, the relationship between acute stress and BDNF is more complex. Acute physical stress in rodents, induced either by physical exercise or foot shock, positively correlated with the induction of BDNF expression, especially in the hippocampus (28–30), a response that can facilitate synaptic plasticity and promote the formation of new neural connections, which are crucial for learning and memory processes (i.e., adaptation). On the other hand, acute psychological or restraint stress inhibits the expression of BDNF in many brain regions, including the hippocampus and the prefrontal cortex (28, 31).

The aforementioned role of BDNF in many psychiatric and neurodegenerative diseases, is tightly correlated to its reciprocal interaction with neuroinflammation (32). BDNF can activate astrocytes and microglia, promoting proinflammatory responses, thus exacerbating neuroinflammation, a process observed in various neurological disorders (33). On the other hand, it’s worth noting that neuroinflammation itself can enhance BDNF signaling and expression in microglia, creating a positive feedback loop (34). Furthermore, activated microglia, can trigger the release of BDNF, adding another layer of complexity to the interplay between this neurotrophin and inflammatory processes in the brain (35). These intricate interactions underscore the significance of understanding how BDNF and neuroinflammation jointly influence the pathophysiology of numerous brain-related conditions.

Given the role that GCs and BDNF seem to possess in stress, brain physiology and stress-related neuropsychiatric disease, and the close association between the expression of GR, BDNF and TrkB across brain regions (36), a reasonable question arises, whether the molecular biology of these two regulatory molecules interact, and if yes, at what level, under which conditions and impacting which cells/cellular networks/dynamics?

## 2 Overview of the GC-BDNF crosstalk

The first publications on the potential interaction between GCs and BDNF emerged in the mid to late 90s, and laid the foundation for our understanding of this intricate relationship. These pioneering investigations revealed that GCs could depress the activity-dependent expression of *Bdnf* mRNA and its receptor, *trkB*, in rodent hippocampal neurons in *in vitro* (37), as well as *in vivo* experiments (38, 39). Building upon this work, Schaaf and colleagues in 1998 and Jacobsen and colleagues in 2006, further demonstrated this phenomenon at the protein level, showing BDNF inhibition in the rat hippocampus following acute and chronic corticosterone administration, respectively (40, 41).

More recent studies have delved even deeper into the effects of GCs on BDNF, examining its different forms. A study by Li et al., showcased that exposure to corticosterone increased the levels of proBDNF (the immature form of BDNF) in the ventral hippocampal dentate gyrus (DG), while simultaneously decreasing the levels of mature BDNF (mBDNF) in both the dorsal and ventral DG, thus significantly diminishing the mBDNF/proBDNF ratio in the hippocampal DG (42). Furthermore, long-term corticosterone administration in mice has been found to increase proBDNF not only in the hippocampal area but also in the cerebellum and the pituitary gland (43). These changes in proBDNF were associated with the development of depressive- and anxiety-like behaviors, suggesting a potential role for proBDNF in the pathogenesis of mood disorders.

Data collected postmortem from subjects suffering from schizophrenia and from a mouse model of the disease, revealed high cortisol and low BDNF levels in serum, prefrontal cortex and cerebrospinal fluid compared to age-matched healthy controls, postulating a role for these molecules in the pathophysiology of schizophrenia (44). HPA axis dysregulation is considered a potent biomarker in many neuropsychiatric disorders, including schizophrenia. Impairment of GC input upon the paraventricular nucleus (PVN) of the hypothalamus has been found to result in a cascade of effects, including an enhancement of CRH expression, an upregulation of hypothalamic levels of BDNF and increased TrkB phosphorylation in the PVN, ultimately leading to the disinhibition of the HPA axis (45). Notably, in a study by Demuyser et al. where corticosterone was chronically released through subcutaneously-implanted pellets in mice, a robust induction of depressive-like behaviors was observed. This depressive-like state was accompanied by a significant decrease in the hippocampal levels of BDNF (46).

The consistent effect of GC-mediated alterations on BDNF expression and the concurrence of increased circulating GC levels and low BDNF in different mood disorders, foreshadows a direct transcriptomic regulatory mechanism. Within the BDNF gene, a putative Glucocorticoid Response Element (GRE) resides in the promoter region IV (47), prompting the hypothesis that the observed GC-mediated repression of BDNF is mediated through its receptor, GR. Substantiating this notion, Hansson et al. employed exon-specific riboprobe *in situ* hybridization to demonstrate that corticosterone administration significantly attenuated exon IV expression in the rat hippocampus (48). Building upon this foundational research, recent studies further elucidated the physical interaction of GR with EGR1 and the direct binding of this receptor-complex to *Bdnf* regulatory sequences within the exon IV promoter, resulting in the repression of transcript 4 expression (49, 50).

Additional compelling evidence implicating the GR in the repression of BDNF was gleaned from *in vivo* investigations. Studies involving GR-impaired mice revealed that, in the presence of stress conditions, dysfunctional GR led to an increase in BDNF expression (51). Moreover, in the context of surgical stress, the phosphorylation of GR by CDK5 was found to diminish BDNF expression, concurrently promoting cognitive dysfunction in aged mice (52). But to further highlight on the interesting complexity of the reciprocal influence between GR and BDNF, Lambert et al. revealed that BDNF administration could also induce GR phosphorylation (53). Notably, this BDNF-mediated phosphorylation, which occurred at distinct amino acids compared to CDK5-mediated phosphorylation, precipitated alterations in the GR transcriptome, ultimately fostering neuronal growth and differentiation.

Meticulous work performed by Revest and colleagues over the past two decades, has identified two more GRE-containing proteins as potential mediators of the GC-BDNF crosstalk: the tissue Plasminogen Activator (tPA) and its inhibitor Plasminogen Activator Inhibitor-1 (PAI-1). Expression of these GC-responsive proteins appears to be regulated in a dose-dependent manner with low to normal GC-secreted levels favoring the expression of tPA (10), while high GC levels, due to intense stress, increase the expression of the inhibitor (54). tPA is responsible for the cleavage of plasminogen to plasmin, which in turn mediates the maturation of proBDNF to mBDNF. The team demonstrated that traumatic stress-related disturbances of the fine balance between tPA and PAI-1 in mice, was enough to impair memory formation and induce PTSD-like memories (54).

Finally, another intriguing aspect of the crosstalk between the two systems, that was presented by Numakawa et al, involves the ability of GR to interact with TrkB in order to promote BDNF-induced glutamate release in neuronal cultures (55). Both GR-TrkB interaction and its subsequent BDNF-mediated signaling was repressed by chronic GC administration.

Ultimately, the aforementioned molecular interactions between the GC-mediated and BDNF-mediated subcellular/cellular systems are translated into behavioral changes. Dysregulation of this intricate balance can precipitate the development of depressive-like behaviors and contribute to the pathogenesis of neuropsychiatric and mood disorders (56). Notably, impaired motivational and forced swim performance due to chronic corticosterone exposure in adult male mice was restored after microinfused replenishment of hippocampal BDNF levels (57). Furthermore, the confluence of BDNF insufficiency and HPA axis dysregulation due to chronic corticosterone exposure has been correlated with heightened amotivation and behavioral despair in murine models (58, 59). Recent studies employing mouse models designed to simulate GC-induced depression and psychotic phenotypes have yielded some noteworthy findings. More specifically, they have demonstrated that the induction of BDNF and/or TrkB activity can significantly alleviate the depressive and psychotic-like behaviors observed in these models, respectively (60, 61).

## 3 GC-BDNF interactions at the neuronal level

**Figure 1:**
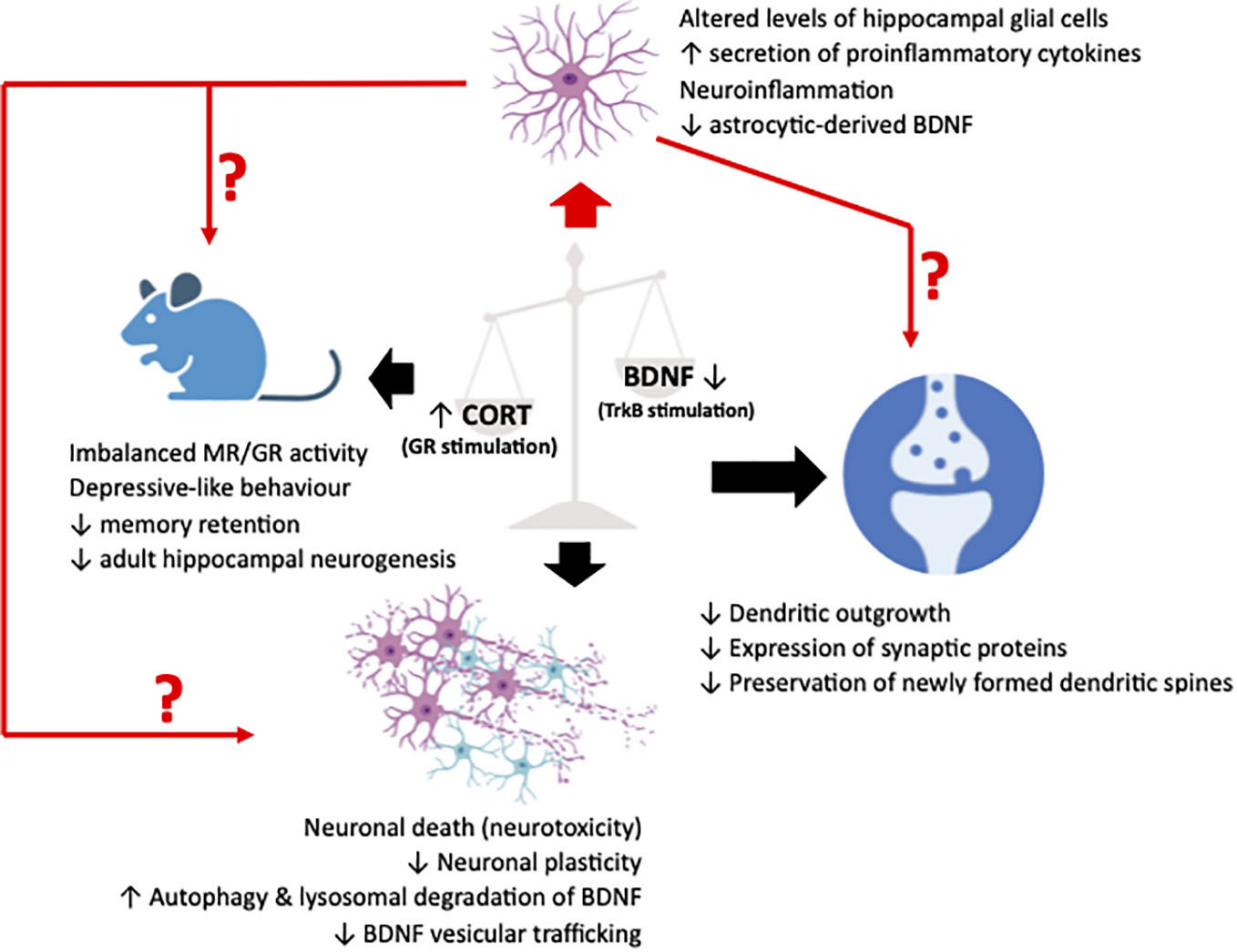
Illustrative representation summarizing the pathological effects in synapses, neurons and cognition/behavior of animal (murine) models (black arrows), resulting from an imbalance between glucocorticoids (CORT) and brain-derived neurotrophic factor (BDNF), leading to an upregulated stimulation of the glucocorticoid receptor (GR) and a downregulated stimulation of the BDNF receptor, TrkB. Recently, neuroinflammatory manifestations due to perturbations in the fine harmonical interaction of the two systems have been described. These manifestations have been associated to alterations in glial physiology and reactivity that could act as a mediator or simply a deteriorating factor of the well-characterized neuronal effects. The figure was created using the BioRender application.

Since the initial delineation of the earliest indications suggesting the existence of a GC-BDNF crosstalk, extensive research efforts have been dedicated to elucidating its role in regulating neuronal physiology and function. This focus is mainly due to BDNF’s well-established role as a key regulator of neuronal growth, survival, and synaptic plasticity, making it a central player in neuronal function and behavior. Consequently, given the profound implications for neuropsychiatric and mood disorders, many studies have diligently focused on elucidating the potential involvement of this interaction in various facets of neuronal viability and function, including synaptic plasticity, the trafficking of BDNF vesicles, and neuronal autophagy (**Figure 1**).

### 3.1 Synaptic plasticity/memory consolidation

Perhaps the most extensively investigated aspect of the effects stemming from the interaction between GCs and BDNF in neurons, pertains to their influence on neuronal plasticity and memory consolidation. The initial evidence for this interplay was provided by Zhou et al., who elucidated corticosterone’s inhibitory impact on the generation of long-term potentiation (LTP) in rat hippocampal slices, an effect that could be counteracted by BDNF administration (62). Presently, it has become evident that the well-defined positive modulatory role of GC-BDNF interaction in several facets of memory formation and learning, necessitates the maintenance of a delicate equilibrium between GC administration and GR activation on one side and BDNF expression and TrkB activation on the other. Prolonged elevation of GC levels and/or compromised neurotrophic support, lead to the development of deficits in spatial memory, contextual fear memory, or fear extinction memory (10, 63, 64).

Mechanistically, recent sources of evidence have unveiled several pathways and molecular processes implemented in GC-BDNF signaling integration that promote synaptic plasticity and memory consolidation. In an *in vitro* setting, the excessive administration of a synthetic GC, dexamethasone (Dex) (a potent GR activator), to immature neurons, exerted inhibitory effects on BDNF-mediated synaptic maturation through two distinct mechanisms: transcriptional repression of synaptic proteins and constriction of their dendritic outgrowth (65). In a study conducted in rats investigating the role of hippocampal GR in memory retention, researchers identified a GR-mediated, non-genomic activation of the CaMKIIα-BDNF-CREB pathway, which was found to be indispensable for rescuing rats from amnesia, induced by GR inhibition (66). Further elucidating on this mechanism, Arango-Lievano et al. demonstrated that neurotrophin priming of GR, preserves GR phosphorylation at BDNF-sensitive sites, guiding GR’s transcriptional machinery and promoting neuronal plasticity (67). Subsequent pioneering work by the same research team, showed that both chronic stress and BDNF deficiency significantly inhibit this BDNF-dependent GR phosphorylation, resulting in impairments in memory retention and post-training dendritic spine preservation (68), increased Tau phosphorylation and development of Tau neuropathology (69), as well as exacerbation of the neuropathological manifestations of the APP/PS1 mouse model of AD (70). Interestingly, in the latter study, using postmortem human prefrontal cortex samples from AD patients, the team was able to positively correlate BDNF-dependent GR phosphorylation with increased cognitive performance, in contrast to the cortisol-dependent GR phosphorylation which was associated with cognitive deficiencies. These results reveal that imbalance between BDNF-mediated and GC-mediated GR phosphorylation in favor of GCs, can contribute to the development and progression of AD dementia.

### 3.2 Neuronal survival

Beyond its role in synaptic plasticity, an excessive imbalance of GC-BDNF interactions, can elicit significantly more deleterious effects on neurons, potentially even threatening their survival. The neuroprotective importance of GC-BDNF interactions has been extensively researched. *In vitro* studies conducted on primary hippocampal neurons and mouse hippocampal cell lines have delineated the neuroprotective potential of BDNF/TrkB/CREB pathway activation in mitigating GC-induced neurotoxicity (71, 72). Additionally, compelling evidence by Zhang et al., experimenting in rats following traumatic brain injury, revealed the contrasting outcomes associated with GR and MR activation (73). High GC levels, inducing GR activation, were found to promote neuronal death in the PVN, while low GC levels, promoting MR activation, supported neuronal survival. These findings underscore the paramount importance of maintaining a balanced GR/MR expression and activation ratio, reflective of a healthy circadian and pulsatile secretory pattern of GCs, as a critical factor for neuroprotection in the face of neurotoxic challenges.

### 3.3 Dendritic BDNF transport

Recently, an intriguing facet of the GC-BDNF interactions has come to light, offering promising prospects for the development of novel therapeutic strategies targeting stress-related disorders. Following translation, BDNF protein within neurons is encapsulated in secretory vesicles and subsequently transported anterogradely to dendrites (74). Adachi et al. characterized the ability of GCs to modulate this transportation process through the upregulation of Huntingtin protein via GR activation (75). In cortical neuronal cultures subjected to a single Dex treatment, HTT-mediated transportation of BDNF-containing vesicles to dendrites was enhanced. Conversely, Agasse et al., while investigating the mechanisms underlying the reduction of adult hippocampal neurogenesis in the context of chronic stress, observed a contrasting effect on GC-mediated BDNF vesicle transportation (76). Specifically, the administration of a very high dose of Dex, mirroring GC circulating levels during chronic stress, within a cortico-hippocampal network configuration, impeded BDNF vesicle transportation. This effect was directly correlated with hyperphosphorylation of HTT. Furthermore, *in vivo* experiments conducted in mice by the same research team replicated the GC-induced HTT phosphorylation and demonstrated a significant suppression of hippocampal neurogenesis as a consequence. The contradictory results observed in these studies are likely attributable to the significant difference in the Dexamethasone dose concentration used in each experiment, highlighting the dose-dependent nature of GC’s influence on BDNF.

### 3.4 Autophagy

Physiological secretion of GCs has been associated with the regulation of neuronal autophagy, acting as a mediator of neuroprotection and initiating the stress-coping response. However, prolonged or excessive exposure to GCs can disrupt the equilibrium of neuronal autophagy, potentially contributing to neuronal dysfunction and neurodegenerative processes (77). In a recently published study conducted by Zhang et al., the research team elucidated how chronic corticosterone exposure induced hyperactivation of the autophagic machinery in neuronal cells within the DG region of the brain (78). This led to an augmented lysosomal degradation of BDNF, which, in conjunction with decreased neuronal proliferation, migration, and survival, ultimately culminated in the development of depressive-like behavior in mice. Additionally, it is noteworthy that GCs have also been implicated in the regulation of the recently characterized pathway known as secretory autophagy, a non-degradative pathway for the extracellular secretion of cytosolic cargo. Specifically, GC-mediated stress has the capability to induce the secretion of matrix metalloproteinase 9 (MMP9) through secretory autophagy, consequently promoting the maturation of BDNF in the extracellular milieu (79).

Altogether, a potential neuronal interplay between the GC/GR and the BDNF/TrkB systems emerges. The previously reviewed studies unveil both the ability of GCs to interfere and alter (mainly through GR) TrkB phosphorylation and BDNF expression and trafficking (37, 45, 48, 49, 75, 76), as well as the ability of BDNF (mainly through TrkB signaling) to prime GR, enabling the maintenance of its phosphorylation even after BDNF is no longer available (67, 68), thus creating a positive feedback loop with two arms, that can promote neuronal plasticity.

## 4 GC-BDNF interactions at the glial level

### 4.1 Glial cells: mediators of stress-induced neuroinflammatory responses

The intricate role of stress-induced neuroinflammation in the development and progression of mood disorders has been the subject of profound scientific inquiry, leading to the accumulation of compelling research evidence (80). From the increased levels of proinflammatory cytokines (81, 82), such as interleukin-6 (IL-6) and tumor necrosis factor-alpha (TNF-α), measured in individuals diagnosed with depression, to the PET scan visualization of neuroinflammation in individuals experiencing depressive episodes (83–85). But the connection between stress and neuroinflammation is not unidirectional; neuroinflammation itself can sensitize the brain to future stressors, perpetuating this vicious cycle, in many ways. Increased levels of the pro-inflammatory cytokine IL-6 can increase the activity of brain regions implicated in the etiology of depression and impair brain connectivity causing mood reduction (86). Furthermore, neuroinflammation can disrupt the balance of neurotransmitters, such as serotonin, in mice, leading to increased vulnerability to stress and mood disorders (87). Another way to impede coping with and recovering from the effects of a future stressor, is through suppression of neuroplasticity, a trait that is well-correlated to chronic inflammation (88).

Glial cells, specifically microglia and astrocytes, play pivotal roles as primary mediators of the aforementioned effects of stress-induced neuroinflammation. Both microgliosis and astrogliosis represent common responses to various stressors, observed in both animal models and individuals with MDD (85, 89). The activation of these glial cells significantly amplifies the release of pro-inflammatory cytokines, intensifying neuroinflammation by disrupting the blood-brain barrier and facilitating the entry of peripheral immune cells and molecules into the brain (90, 91). Concurrently, activated glial cells contribute to neurodegeneration and hinder synaptic plasticity. Clinical and postmortem studies have well-documented the association between astrocytic dysfunction and depressive-like phenotypes (92). Moreover, morphological and functional atrophy of astroglial cells has been shown to induce depressive-like phenotypes in animal models.

### 4.2 Novel research on GC-BDNF interaction in glial cells

Although it becomes evident that potential effects in non-neuronal cells could be in existence, with crucial role in maintaining brain homeostasis and responding to neuroinflammatory challenges, the predominant focus of the existing literature on GC-BDNF interactions, as previously demonstrated, relates to their effects on neurons. This trend in the field is inconsistent with the impressively high GR/MR ratio in glial cells compared to neurons (93–96), with single-cell RNA-seq data extending this observation in gene expression to known GR and MR target genes (97). As a result, non-neuronal cell populations are significantly more sensitive to both normal (i.e. at the peaks of ultradian GC secretion) and abnormal GC alterations (e.g. under stress). Similar cell type specific expression profiles have been characterized in the BDNF/TrkB system as well, with BDNF basal expression levels significantly elevated in pyramidal neurons compared to TrkB, while inhibitory neuronal and glial populations mainly expressing the TrkB receptor (98). Such a variety in the proportionate expression between the components of the two systems, suggests potential differences in the glucocorticoid response not just between neuronal and non-neuronal populations but even within the different glial cell types.

Several recent studies have characterized a co-occurrence of repressed BDNF expression and altered levels of microglia and astrocytes in the hippocampus of rats exposed to chronic stress, accompanied by increased secretion of pro-inflammatory cytokines (99–101). However, these studies have not showcased any evidence of a direct correlation between BDNF and GCs. In another work by Chen et al., adrenalectomized rats exposed to chronic mild stress exhibited astrocytic dysfunction in prefrontal cortex. This impairment was associated with reduced BDNF levels in the hippocampus and correlated with anxiety-like behavior displayed by the animals (102). Nevertheless, neither this study delved into investigating the potential linkage between the impaired GC-BDNF interaction and astrocyte dysfunction.

Recent work in our laboratory attempted to decipher the acute effects of GC administration on BDNF expression in primary astrocytic cultures (103). Astrocytes were able to instantly respond to a physiological GC pulse in their environment in a biphasic manner, in which BDNF expression was upregulated within the first hour, followed by a GR-dependent, downregulated BDNF expression for the rest of the 24-hour cycle. Additionally, in an interesting demonstration of GC-mediated homeostatic capabilities, astrocytes exposed to a second identical GC pulse within the same circadian cycle, were able to normalize their BDNF expression levels. These findings highlight the neuroprotective importance of maintaining a normal ultradian rhythmicity and the potential devastating effects that stress-related deregulation of it, can have in the astrocyte-mediated trophic support of the surrounding neurons. Furthermore, our results demonstrate astrocyte’s ability to alter their responses to increased GC secretion based on whether this increase is transient or consistent, an effect that can be attributed to their increased, GR-mediated GC sensitivity.

Overall, the aforementioned evidence of stress-induced neuroinflammation mediated through glial cells, the co-occurrence of irregularities in glial pathology and BDNF repression in chronically stressed mice and the existence of an astrocytic, GC-induced, biphasic response in BDNF expression and TrkB activation (103), unveils a new dimension in the interplay between the two systems as effectors of glial physiology. Such glial effects could subsequently deteriorate or even mediate the detrimental effects described in neuronal populations (**Figure 1**). Combining this with the emerging role of non-neuronal cells in neuronal support, plasticity and synapse formation, arises the need to further investigate and fully unravel the GC-BDNF interaction in these cell populations. The results of these studies could pave the way for new therapeutic approaches targeting stress-related neurodegenerative disorders.

## 5 Clinical significance of the GC-BDNF interactions

The interaction of GCs with BDNF may have clinical implications given the role of both molecules in the regulation of human behavior, mood and their involvement in neuropsychiatric disease. BDNF modulates the efficacy of glutamatergic neurotransmission through controlling the number of synapses or spines, the amount of transmitter release, and receptor expression or function, preventing synaptic fatigue and facilitating synaptic plasticity, thus implicated in higher order cognitive processing (104). GCs also regulate glutamate release as well as the expression of glutamatergic receptors by neurons (105). Moreover, both molecules modulate neurogenic events in hippocampus, linked to changes in cognitive and behavioral phenotypes (106, 107), including efficacy of many antidepressant drugs (108). Mood disorders (and in particular depression) are linked to changes in glutamate release, homeostasis of the glutamine-glutamate-energy production cycle, expression of glutamatergic receptors and their functional interplay with glutamate, leading to modifications in synaptic plasticity, dendritic remodeling and glutamate excitotoxicity (inducing neuroinflammation and reduced cellular viability) (105, 109). Moreover, another key phenomenon of depression is the disrupted neurogenic capacity in hippocampus, linked to the cognitive and behavioral defects, which is also a marker of the efficacy of most major antidepressant drugs (110). Changes in the characteristics of the GC pulsatility have been shown to impose behavioral alterations and changes in the neural processing in healthy subjects (8) as well patients receiving hydrocortisone replacement therapy (111). These alterations refer to the emotional control of cognition and are very relevant to mood regulation. Moreover, disruptions in the physiological stimulation by BDNF (like in the case of the relatively common gene polymorphism V66M) have been also shown to cause susceptibility to mood disorders, and are associated with smaller hippocampal volumes and cognitive impairment (112). Thus, optimal temporal dynamics in the complex interactions of GCs and BDNF might be important for brain homeostasis (for example determining the contextual efficacy of BDNF stimulation), and dysrhythmicity may contribute to the pathophysiology of depression. These approaches could open new experimental pathways in the chrono-pharmacology of mood disorders.

## 6 Conclusion

In essence, when viewed through a specialized lens, glucocorticoids in the brain emerge as orchestrators of a finely-tuned symphony. Their importance extends beyond mere stress response, encompassing intricate temporal dynamics and cellular interactions that influence neural function in profound ways. Understanding this specialized facet of glucocorticoid neurobiology is fundamental for unraveling the subtleties of brain function and dysfunction, with implications for neurodegenerative diseases, mood disorders, and cognitive enhancement strategies. In this regard, BDNF regulation becomes relevant, as it mediates some of the GC effects, while opposing others, possibly in a time-, context- and cell type-dependent manner.

## Author contributions

AT: Investigation, Methodology, Writing – original draft. KK: Conceptualization, Methodology, Validation, Writing – review & editing. IC: Conceptualization, Funding acquisition, Supervision, Validation, Writing – review & editing.

## References

[1] Gjerstad JK Lightman SL Spiga F. Role of glucocorticoid negative feedback in the regulation of HPA axis pulsatility. Stress. (2018) 21:403–16. doi: 10.1080/10253890.2018.1470238 PMC6220752 29764284

[2] Dunlop BW Wong A. The hypothalamic-pituitary-adrenal axis in PTSD: Pathophysiology and treatment interventions. Prog Neuropsychopharmacol Biol Psychiatry. (2019) 89:361–79. doi: 10.1016/j.pnpbp.2018.10.010 30342071

[3] Lamers F Vogelzangs N Merikangas KR de Jonge P Beekman AT Penninx BW. Evidence for a differential role of HPA-axis function, inflammation and metabolic syndrome in melancholic versus atypical depression. Mol Psychiatry. (2013) 18:692–9. doi: 10.1038/mp.2012.144 23089630

[4] Gray JD Kogan JF Marrocco J McEwen BS. Genomic and epigenomic mechanisms of glucocorticoids in the brain. Nat Rev Endocrinol. (2017) 13:661–73. doi: 10.1038/nrendo.2017.97 28862266

[5] Myers B McKlveen JM Herman JP. Glucocorticoid actions on synapses, circuits, and behavior: implications for the energetics of stress. Front Neuroendocrinol. (2014) 35:180–96. doi: 10.1016/j.yfrne.2013.12.003 PMC4422101 24361584

[6] Watts AG Tanimura S Sanchez-Watts G. Corticotropin-releasing hormone and arginine vasopressin gene transcription in the hypothalamic paraventricular nucleus of unstressed rats: daily rhythms and their interactions with corticosterone. Endocrinology. (2004) 145:529–40. doi: 10.1210/en.2003-0394 14563696

[7] Dickmeis T. Glucocorticoids and the circadian clock. J Endocrinol. (2009) 200:3–22. doi: 10.1677/JOE-08-0415 18971218

[8] Kalafatakis K Russell GM Harmer CJ Munafo MR Marchant N Wilson A. Ultradian rhythmicity of plasma cortisol is necessary for normal emotional and cognitive responses in man. Proc Natl Acad Sci U.S.A. (2018) 115:E4091–E100. doi: 10.1073/pnas.1714239115 PMC5924881 29632168

[9] Liston C Cichon JM Jeanneteau F Jia Z Chao MV Gan WB. Circadian glucocorticoid oscillations promote learning-dependent synapse formation and maintenance. Nat Neurosci. (2013) 16:698–705. doi: 10.1038/nn.3387 23624512 PMC3896394

[10] Revest JM Le Roux A Roullot-Lacarriere V Kaouane N Vallee M Kasanetz F. BDNF-TrkB signaling through Erk1/2 MAPK phosphorylation mediates the enhancement of fear memory induced by glucocorticoids. Mol Psychiatry. (2014) 19:1001–9. doi: 10.1038/mp.2013.134 PMC4195976 24126929

[11] Kalafatakis K Russell GM Lightman SL. MECHANISMS IN ENDOCRINOLOGY: Does circadian and ultradian glucocorticoid exposure affect the brain? Eur J Endocrinol. (2019) 180:R73–89. doi: 10.1530/EJE-18-0853 30481157

[12] Ronchetti S Ayroldi E Ricci E Gentili M Migliorati G Riccardi C. A glance at the use of glucocorticoids in rare inflammatory and autoimmune diseases: still an indispensable pharmacological tool? Front Immunol. (2020) 11:613435. doi: 10.3389/fimmu.2020.613435 33584696 PMC7874096

[13] Zou HJ Guo SW Zhu L Xu X Liu JB. Methylprednisolone induces neuro-protective effects *via* the inhibition of A1 astrocyte activation in traumatic spinal cord injury mouse models. Front Neurosci. (2021) 15:628917. doi: 10.3389/fnins.2021.628917 34135725 PMC8200570

[14] Ryan KM Boyle NT Harkin A Connor TJ. Dexamethasone attenuates inflammatory-mediated suppression of beta(2)-adrenoceptor expression in rat primary mixed glia. J Neuroimmunol. (2020) 338:577082. doi: 10.1016/j.jneuroim.2019.577082 31707103

[15] Piechota M Korostynski M Golda S Ficek J Jantas D Barbara Z. Transcriptional signatures of steroid hormones in the striatal neurons and astrocytes. BMC Neurosci. (2017) 18:37. doi: 10.1186/s12868-017-0352-5 28381250 PMC5381047

[16] Maturana CJ Aguirre A Saez JC. High glucocorticoid levels during gestation activate the inflammasome in hippocampal oligodendrocytes of the offspring. Dev Neurobiol. (2017) 77:625–42. doi: 10.1002/dneu.22409 27314460

[17] Frank MG Hershman SA Weber MD Watkins LR Maier SF. Chronic exposure to exogenous glucocorticoids primes microglia to pro-inflammatory stimuli and induces NLRP3 mRNA in the hippocampus. Psychoneuroendocrinology. (2014) 40:191–200. doi: 10.1016/j.psyneuen.2013.11.006 24485491 PMC3912460

[18] Dey A Hao S Erion JR Wosiski-Kuhn M Stranahan AM. Glucocorticoid sensitization of microglia in a genetic mouse model of obesity and diabetes. J Neuroimmunol. (2014) 269:20–7. doi: 10.1016/j.jneuroim.2014.01.013 PMC3989932 24534266

[19] Wyss-Coray T Mucke L. Inflammation in neurodegenerative disease–a double-edged sword. Neuron. (2002) 35:419–32. doi: 10.1016/S0896-6273(02)00794-8 12165466

[20] Zuccato C Cattaneo E. Brain-derived neurotrophic factor in neurodegenerative diseases. Nat Rev Neurol. (2009) 5:311–22. doi: 10.1038/nrneurol.2009.54 19498435

[21] Gao L Zhang Y Sterling K Song W. Brain-derived neurotrophic factor in Alzheimer's disease and its pharmaceutical potential. Transl Neurodegener. (2022) 11:4. doi: 10.1186/s40035-022-00279-0 35090576 PMC8796548

[22] Wang ZH Xiang J Liu X Yu SP Manfredsson FP Sandoval IM. Deficiency in BDNF/trkB neurotrophic activity stimulates delta-secretase by upregulating C/EBPbeta in alzheimer's disease. Cell Rep. (2019) 28:655–69 e5. doi: 10.1016/j.celrep.2019.06.054 31315045 PMC6684282

[23] Jiao SS Shen LL Zhu C Bu XL Liu YH Liu CH. Brain-derived neurotrophic factor protects against tau-related neurodegeneration of Alzheimer's disease. Transl Psychiatry. (2016) 6:e907. doi: 10.1038/tp.2016.186 27701410 PMC5315549

[24] Kim HI Lee S Lim J Chung S Koo TS Ji YG. ERRgamma ligand HPB2 upregulates BDNF-TrkB and enhances dopaminergic neuronal phenotype. Pharmacol Res. (2021) 165:105423. doi: 10.1016/j.phrs.2021.105423 33434621

[25] Notaras M van den Buuse M. Neurobiology of BDNF in fear memory, sensitivity to stress, and stress-related disorders. Mol Psychiatry. (2020) 25:2251–74. doi: 10.1038/s41380-019-0639-2 31900428

[26] Cheng S Wang W Zhu Z Zhao M Li H Liu D. Involvement of brain-derived neurotrophic factor methylation in the prefrontal cortex and hippocampus induced by chronic unpredictable mild stress in male mice. J Neurochem. (2023) 164:624–42. doi: 10.1111/jnc.15735 36453259

[27] Niknazar S Nahavandi A Peyvandi AA Peyvandi H Akhtari AS Karimi M. Comparison of the adulthood chronic stress effect on hippocampal BDNF signaling in male and female rats. Mol Neurobiol. (2016) 53:4026–33. doi: 10.1007/s12035-015-9345-5 26189832

[28] Ieraci A Mallei A Musazzi L Popoli M. Physical exercise and acute restraint stress differentially modulate hippocampal brain-derived neurotrophic factor transcripts and epigenetic mechanisms in mice. Hippocampus. (2015) 25:1380–92. doi: 10.1002/hipo.22458 25820928

[29] Franks H Wang R Li M Wang B Wildmann A Ortyl T. Heat shock factor HSF1 regulates BDNF gene promoters upon acute stress in the hippocampus, together with pCREB. J Neurochem. (2023) 165:131–48. doi: 10.1111/jnc.15707 PMC10097844 36227087

[30] Rojas Vega S Struder HK Vera Wahrmann B Schmidt A Bloch W Hollmann W. Acute BDNF and cortisol response to low intensity exercise and following ramp incremental exercise to exhaustion in humans. Brain Res. (2006) 1121:59–65. doi: 10.1016/j.brainres.2006.08.105 17010953

[31] Li G Wang Y Yan M Ma H Gao Y Li Z. Time-dependent co-relation of BDNF and CREB mRNAs in adult rat brains following acute psychological stress in the communication box paradigm. Neurosci Lett. (2016) 624:34–41. doi: 10.1016/j.neulet.2016.04.039 27132084

[32] Lima Giacobbo B Doorduin J Klein HC Dierckx R Bromberg E de Vries EFJ. Brain-derived neurotrophic factor in brain disorders: focus on neuroinflammation. Mol Neurobiol. (2019) 56:3295–312. doi: 10.1007/s12035-018-1283-6 PMC6476855 30117106

[33] Ding H Chen J Su M Lin Z Zhan H Yang F. BDNF promotes activation of astrocytes and microglia contributing to neuroinflammation and mechanical allodynia in cyclophosphamide-induced cystitis. J Neuroinflamm. (2020) 17:19. doi: 10.1186/s12974-020-1704-0 PMC6958761 31931832

[34] Lai SW Chen JH Lin HY Liu YS Tsai CF Chang PC. Regulatory effects of neuroinflammatory responses through brain-derived neurotrophic factor signaling in microglial cells. Mol Neurobiol. (2018) 55:7487–99. doi: 10.1007/s12035-018-0933-z 29427085

[35] Gomes C Ferreira R George J Sanches R Rodrigues DI Goncalves N. Activation of microglial cells triggers a release of brain-derived neurotrophic factor (BDNF) inducing their proliferation in an adenosine A2A receptor-dependent manner: A2A receptor blockade prevents BDNF release and proliferation of microglia. J Neuroinflamm. (2013) 10:16. doi: 10.1186/1742-2094-10-16 PMC3567964 23363775

[36] Kalafatakis K Giannakeas N Lightman SL Charalampopoulos I Russell GM Tsipouras M. Utilization of the allen gene expression atlas to gain further insight into glucocorticoid physiology in the adult mouse brain. Neurosci Lett. (2019) 706:194–200. doi: 10.1016/j.neulet.2019.05.020 31100428

[37] Cosi C Spoerri PE Comelli MC Guidolin D Skaper SD. Glucocorticoids depress activity-dependent expression of BDNF mRNA in hippocampal neurones. Neuroreport. (1993) 4:527–30. doi: 10.1097/00001756-199305000-00016 8513132

[38] Schaaf MJ Hoetelmans RW de Kloet ER Vreugdenhil E. Corticosterone regulates expression of BDNF and trkB but not NT-3 and trkC mRNA in the rat hippocampus. J Neurosci Res. (1997) 48:334–41. doi: 10.1002/(SICI)1097-4547(19970515)48:4<334::AID-JNR5>3.0.CO;2-C 9169859

[39] Smith MA Makino S Kvetnansky R Post RM. Stress and glucocorticoids affect the expression of brain-derived neurotrophic factor and neurotrophin-3 mRNAs in the hippocampus. J Neurosci. (1995) 15:1768–77. doi: 10.1523/JNEUROSCI.15-03-01768.1995 PMC6578156 7891134

[40] Schaaf MJ de Jong J de Kloet ER Vreugdenhil E. Downregulation of BDNF mRNA and protein in the rat hippocampus by corticosterone. Brain Res. (1998) 813:112–20. doi: 10.1016/S0006-8993(98)01010-5 9824681

[41] Jacobsen JP Mork A. Chronic corticosterone decreases brain-derived neurotrophic factor (BDNF) mRNA and protein in the hippocampus, but not in the frontal cortex, of the rat. Brain Res. (2006) 1110:221–5. doi: 10.1016/j.brainres.2006.06.077 16876769

[42] Li J Chen J Ma N Yan D Wang Y Zhao X. Effects of corticosterone on the expression of mature brain-derived neurotrophic factor (mBDNF) and proBDNF in the hippocampal dentate gyrus. Behav Brain Res. (2019) 365:150–6. doi: 10.1016/j.bbr.2019.03.010 30851317

[43] Lin L Herselman MF Zhou XF Bobrovskaya L. Effects of corticosterone on BDNF expression and mood behaviours in mice. Physiol Behav. (2022) 247:113721. doi: 10.1016/j.physbeh.2022.113721 35074305

[44] Issa G Wilson C Terry AV Jr. Pillai A. An inverse relationship between cortisol and BDNF levels in schizophrenia: data from human postmortem and animal studies. Neurobiol Dis. (2010) 39:327–33. doi: 10.1016/j.nbd.2010.04.017 20451611

[45] Jeanneteau FD Lambert WM Ismaili N Bath KG Lee FS Garabedian MJ. BDNF and glucocorticoids regulate corticotrophin-releasing hormone (CRH) homeostasis in the hypothalamus. Proc Natl Acad Sci U.S.A. (2012) 109:1305–10. doi: 10.1073/pnas.1114122109 PMC3268297 22232675

[46] Demuyser T Bentea E Deneyer L Albertini G Massie A Smolders I. Disruption of the HPA-axis through corticosterone-release pellets induces robust depressive-like behavior and reduced BDNF levels in mice. Neurosci Lett. (2016) 626:119–25. doi: 10.1016/j.neulet.2016.05.026 27208833

[47] Timmusk T Palm K Metsis M Reintam T Paalme V Saarma M. Multiple promoters direct tissue-specific expression of the rat BDNF gene. Neuron. (1993) 10:475–89. doi: 10.1016/0896-6273(93)90335-O 8461137

[48] Hansson AC Sommer WH Metsis M Stromberg I Agnati LF Fuxe K. Corticosterone actions on the hippocampal brain-derived neurotrophic factor expression are mediated by exon IV promoter. J Neuroendocrinol. (2006) 18:104–14. doi: 10.1111/j.1365-2826.2005.01390.x 16420279

[49] Chen H Lombes M Le Menuet D. Glucocorticoid receptor represses brain-derived neurotrophic factor expression in neuron-like cells. Mol Brain. (2017) 10:12. doi: 10.1186/s13041-017-0295-x 28403881 PMC5389111

[50] Chen H Amazit L Lombes M Le Menuet D. Crosstalk between glucocorticoid receptor and early-growth response protein 1 accounts for repression of brain-derived neurotrophic factor transcript 4 expression. Neuroscience. (2019) 399:12–27. doi: 10.1016/j.neuroscience.2018.12.012 30578973

[51] Alboni S Tascedda F Corsini D Benatti C Caggia F Capone G. Stress induces altered CRE/CREB pathway activity and BDNF expression in the hippocampus of glucocorticoid receptor-impaired mice. Neuropharmacology. (2011) 60:1337–46. doi: 10.1016/j.neuropharm.2011.01.050 21324325

[52] Tian XS Tong YW Li ZQ Li LX Zhang T Ren TY. Surgical stress induces brain-derived neurotrophic factor reduction and postoperative cognitive dysfunction *via* glucocorticoid receptor phosphorylation in aged mice. CNS Neurosci Ther. (2015) 21:398–409. doi: 10.1111/cns.12368 25611431 PMC6495659

[53] Lambert WM Xu CF Neubert TA Chao MV Garabedian MJ Jeanneteau FD. Brain-derived neurotrophic factor signaling rewrites the glucocorticoid transcriptome *via* glucocorticoid receptor phosphorylation. Mol Cell Biol. (2013) 33:3700–14. doi: 10.1128/MCB.00150-13 PMC3753865 23878391

[54] Bouarab C Roullot-Lacarriere V Vallee M Le Roux A Guette C Mennesson M. PAI-1 protein is a key molecular effector in the transition from normal to PTSD-like fear memory. Mol Psychiatry. (2021) 26:4968–81. doi: 10.1038/s41380-021-01024-1 PMC8589667 33510345

[55] Numakawa T Kumamaru E Adachi N Yagasaki Y Izumi A Kunugi H. Glucocorticoid receptor interaction with TrkB promotes BDNF-triggered PLC-gamma signaling for glutamate release *via* a glutamate transporter. Proc Natl Acad Sci U.S.A. (2009) 106:647–52. doi: 10.1073/pnas.0800888106 PMC2626757 19126684

[56] Notaras M Hill R Gogos JA van den Buuse M. BDNF Val66Met genotype determines hippocampus-dependent behavior *via* sensitivity to glucocorticoid signaling. Mol Psychiatry. (2016) 21:730–2. doi: 10.1038/mp.2015.152 PMC4879187 26821977

[57] Gourley SL Kiraly DD Howell JL Olausson P Taylor JR. Acute hippocampal brain-derived neurotrophic factor restores motivational and forced swim performance after corticosterone. Biol Psychiatry. (2008) 64:884–90. doi: 10.1016/j.biopsych.2008.06.016 PMC2633780 18675955

[58] Notaras M Du X Gogos J van den Buuse M Hill RA. The BDNF Val66Met polymorphism regulates glucocorticoid-induced corticohippocampal remodeling and behavioral despair. Transl Psychiatry. (2017) 7:e1233. doi: 10.1038/tp.2017.205 28926000 PMC5639248

[59] Gourley SL Swanson AM Jacobs AM Howell JL Mo M Dileone RJ. Action control is mediated by prefrontal BDNF and glucocorticoid receptor binding. Proc Natl Acad Sci U.S.A. (2012) 109:20714–9. doi: 10.1073/pnas.1208342109 PMC3528547 23185000

[60] Lee YJ Kim HR Lee CY Hyun SA Ko MY Lee BS. 2-phenylethylamine (PEA) ameliorates corticosterone-induced depression-like phenotype via the BDNF/trkB/CREB signaling pathway. Int J Mol Sci. (2020) 21. doi: 10.3390/ijms21239103 PMC7729630 33265983

[61] Chen Y Li S Zhang T Yang F Lu B. Corticosterone antagonist or TrkB agonist attenuates schizophrenia-like behavior in a mouse model combining Bdnf-e6 deficiency and developmental stress. iScience. (2022) 25:104609. doi: 10.1016/j.isci.2022.104609 35789832 PMC9250029

[62] Zhou J Zhang F Zhang Y. Corticosterone inhibits generation of long-term potentiation in rat hippocampal slice: involvement of brain-derived neurotrophic factor. Brain Res. (2000) 885:182–91. doi: 10.1016/S0006-8993(00)02934-6 11102572

[63] Gururajan A Hill RA van den Buuse M. Brain-derived neurotrophic factor heterozygous mutant rats show selective cognitive changes and vulnerability to chronic corticosterone treatment. Neuroscience. (2015) 284:297–310. doi: 10.1016/j.neuroscience.2014.10.009 25445195

[64] Klug M Hill RA Choy KH Kyrios M Hannan AJ van den Buuse M. Long-term behavioral and NMDA receptor effects of young-adult corticosterone treatment in BDNF heterozygous mice. Neurobiol Dis. (2012) 46:722–31. doi: 10.1016/j.nbd.2012.03.015 22426399

[65] Kumamaru E Numakawa T Adachi N Yagasaki Y Izumi A Niyaz M. Glucocorticoid prevents brain-derived neurotrophic factor-mediated maturation of synaptic function in developing hippocampal neurons through reduction in the activity of mitogen-activated protein kinase. Mol Endocrinol. (2008) 22:546–58. doi: 10.1210/me.2007-0264 PMC5419617 18096693

[66] Chen DY Bambah-Mukku D Pollonini G Alberini CM. Glucocorticoid receptors recruit the CaMKIIalpha-BDNF-CREB pathways to mediate memory consolidation. Nat Neurosci. (2012) 15:1707–14. doi: 10.1038/nn.3266 PMC3509234 23160045

[67] Arango-Lievano M Lambert WM Bath KG Garabedian MJ Chao MV Jeanneteau F. Neurotrophic-priming of glucocorticoid receptor signaling is essential for neuronal plasticity to stress and antidepressant treatment. Proc Natl Acad Sci U.S.A. (2015) 112:15737–42. doi: 10.1073/pnas.1509045112 PMC4697403 26630005

[68] Arango-Lievano M Borie AM Dromard Y Murat M Desarmenien MG Garabedian MJ. Persistence of learning-induced synapses depends on neurotrophic priming of glucocorticoid receptors. Proc Natl Acad Sci U.S.A. (2019) 116:13097–106. doi: 10.1073/pnas.1903203116 PMC6601006 31182610

[69] Arango-Lievano M Peguet C Catteau M Parmentier ML Wu S Chao MV. Deletion of neurotrophin signaling through the glucocorticoid receptor pathway causes tau neuropathology. Sci Rep. (2016) 6:37231. doi: 10.1038/srep37231 27849045 PMC5110980

[70] Dromard Y Arango-Lievano M Borie A Dedin M Fontanaud P Torrent J. Loss of glucocorticoid receptor phosphorylation contributes to cognitive and neurocentric damages of the amyloid-beta pathway. Acta Neuropathol Commun. (2022) 10:91. doi: 10.1186/s40478-022-01396-7 35733193 PMC9219215

[71] Nitta A Ohmiya M Sometani A Itoh M Nomoto H Furukawa Y. Brain-derived neurotrophic factor prevents neuronal cell death induced by corticosterone. J Neurosci Res. (1999) 57:227–35. doi: 10.1002/(SICI)1097-4547(19990715)57:2<227::AID-JNR8>3.0.CO;2-E 10398300

[72] Mo F Tang Y Du P Shen Z Yang J Cai M. GPR39 protects against corticosterone-induced neuronal injury in hippocampal cells through the CREB-BDNF signaling pathway. J Affect Disord. (2020) 272:474–84. doi: 10.1016/j.jad.2020.03.137 32553391

[73] Zhang B Bai M Xu X Yang M Niu F Gao F. Corticosteroid receptor rebalancing alleviates critical illness-related corticosteroid insufficiency after traumatic brain injury by promoting paraventricular nuclear cell survival *via* Akt/CREB/BDNF signaling. J Neuroinflamm. (2020) 17:318. doi: 10.1186/s12974-020-02000-2 PMC7586672 33100225

[74] Adachi N Kohara K Tsumoto T. Difference in trafficking of brain-derived neurotrophic factor between axons and dendrites of cortical neurons, revealed by live-cell imaging. BMC Neurosci. (2005) 6:42. doi: 10.1186/1471-2202-6-42 15969745 PMC1180452

[75] Adachi N Numakawa T Nakajima S Fukuoka M Odaka H Katanuma Y. Glucocorticoid affects dendritic transport of BDNF-containing vesicles. Sci Rep. (2015) 5:12684. doi: 10.1038/srep12684 26239075 PMC4523857

[76] Agasse F Mendez-David I Christaller W Carpentier R Braz BY David DJ. Chronic corticosterone elevation suppresses adult hippocampal neurogenesis by hyperphosphorylating huntingtin. Cell Rep. (2020) 32:107865. doi: 10.1016/j.celrep.2020.107865 32640230

[77] Jung S Choe S Woo H Jeong H An HK Moon H. Autophagic death of neural stem cells mediates chronic stress-induced decline of adult hippocampal neurogenesis and cognitive deficits. Autophagy. (2020) 16:512–30. doi: 10.1080/15548627.2019.1630222 PMC6999625 31234698

[78] Zhang K Wang F Zhai M He M Hu Y Feng L. Hyperactive neuronal autophagy depletes BDNF and impairs adult hippocampal neurogenesis in a corticosterone-induced mouse model of depression. Theranostics. (2023) 13:1059–75. doi: 10.7150/thno.81067 PMC9925310 36793868

[79] Martinelli S Anderzhanova EA Bajaj T Wiechmann S Dethloff F Weckmann K. Stress-primed secretory autophagy promotes extracellular BDNF maturation by enhancing MMP9 secretion. Nat Commun. (2021) 12:4643. doi: 10.1038/s41467-021-24810-5 34330919 PMC8324795

[80] Troubat R Barone P Leman S Desmidt T Cressant A Atanasova B. Neuroinflammation and depression: A review. Eur J Neurosci. (2021) 53:151–71. doi: 10.1111/ejn.14720 32150310

[81] Kim YK Na KS Shin KH Jung HY Choi SH Kim JB. Cytokine imbalance in the pathophysiology of major depressive disorder. Prog Neuropsychopharmacol Biol Psychiatry. (2007) 31:1044–53. doi: 10.1016/j.pnpbp.2007.03.004 17433516

[82] Dowlati Y Herrmann N Swardfager W Liu H Sham L Reim EK. A meta-analysis of cytokines in major depression. Biol Psychiatry. (2010) 67:446–57. doi: 10.1016/j.biopsych.2009.09.033 20015486

[83] Moraga-Amaro R Guerrin CGJ Reali Nazario L Lima Giacobbo B RA JOD Stehberg J. A single dose of ketamine cannot prevent protracted stress-induced anhedonia and neuroinflammation in rats. Stress. (2022) 25:145–55. doi: 10.1080/10253890.2022.2045269 35384793

[84] Li H Sagar AP Keri S. Translocator protein (18kDa TSPO) binding, a marker of microglia, is reduced in major depression during cognitive-behavioral therapy. Prog Neuropsychopharmacol Biol Psychiatry. (2018) 83:1–7. doi: 10.1016/j.pnpbp.2017.12.011 29269262

[85] Holmes SE Hinz R Conen S Gregory CJ Matthews JC Anton-Rodriguez JM. Elevated translocator protein in anterior cingulate in major depression and a role for inflammation in suicidal thinking: A positron emission tomography study. Biol Psychiatry. (2018) 83:61–9. doi: 10.1016/j.biopsych.2017.08.005 28939116

[86] Harrison NA Brydon L Walker C Gray MA Steptoe A Critchley HD. Inflammation causes mood changes through alterations in subgenual cingulate activity and mesolimbic connectivity. Biol Psychiatry. (2009) 66:407–14. doi: 10.1016/j.biopsych.2009.03.015 PMC2885494 19423079

[87] Hersey M Reneaux M Berger SN Mena S Buchanan AM Ou Y. A tale of two transmitters: serotonin and histamine as in *vivo* biomarkers of chronic stress in mice. J Neuroinflamm. (2022) 19:167. doi: 10.1186/s12974-022-02508-9 PMC9235270 35761344

[88] Golia MT Poggini S Alboni S Garofalo S Ciano Albanese N Viglione A. Interplay between inflammation and neural plasticity: Both immune activation and suppression impair LTP and BDNF expression. Brain Behav Immun. (2019) 81:484–94. doi: 10.1016/j.bbi.2019.07.003 31279682

[89] Ali T Rahman SU Hao Q Li W Liu Z Ali Shah F. Melatonin prevents neuroinflammation and relieves depression by attenuating autophagy impairment through FOXO3a regulation. J Pineal Res. (2020) 69:e12667. doi: 10.1111/jpi.12667 32375205

[90] Zhao D Xu X Pan L Zhu W Fu X Guo L. Pharmacologic activation of cholinergic alpha7 nicotinic receptors mitigates depressive-like behavior in a mouse model of chronic stress. J Neuroinflamm. (2017) 14:234. doi: 10.1186/s12974-017-1007-2 PMC5712092 29197398

[91] Leng L Zhuang K Liu Z Huang C Gao Y Chen G. Menin deficiency leads to depressive-like behaviors in mice by modulating astrocyte-mediated neuroinflammation. Neuron. (2018) 100:551–63 e7. doi: 10.1016/j.neuron.2018.08.031 30220511

[92] Wang Q Jie W Liu JH Yang JM Gao TM. An astroglial basis of major depressive disorder? An overview. Glia. (2017) 65:1227–50. doi: 10.1002/glia.23143 28317185

[93] Vielkind U Walencewicz A Levine JM Bohn MC. Type II glucocorticoid receptors are expressed in oligodendrocytes and astrocytes. J Neurosci Res. (1990) 27:360–73. doi: 10.1002/jnr.490270315 2097380

[94] Groyer G Eychenne B Girard C Rajkowski K Schumacher M Cadepond F. Expression and functional state of the corticosteroid receptors and 11 beta-hydroxysteroid dehydrogenase type 2 in Schwann cells. Endocrinology. (2006) 147:4339–50. doi: 10.1210/en.2005-1625 16763064

[95] Sierra A Gottfried-Blackmore A Milner TA McEwen BS Bulloch K. Steroid hormone receptor expression and function in microglia. Glia. (2008) 56:659–74. doi: 10.1002/glia.20644 18286612

[96] Shaqura M Li X Al-Madol MA Tafelski S Beyer-Koczorek A Mousa SA. Acute mechanical sensitization of peripheral nociceptors by aldosterone through non-genomic activation of membrane bound mineralocorticoid receptors in naive rats. Neuropharmacology. (2016) 107:251–61. doi: 10.1016/j.neuropharm.2016.03.032 27016023

[97] Viho EMG Buurstede JC Berkhout JB Mahfouz A Meijer OC. Cell type specificity of glucocorticoid signaling in the adult mouse hippocampus. J Neuroendocrinol. (2022) 34:e13072. doi: 10.1111/jne.13072 34939259 PMC9286676

[98] Jeanneteau F. Fast signaling by glucocorticoids shapes neural representations of behaviors. Steroids. (2023) 199:109294. doi: 10.1016/j.steroids.2023.109294 37549777

[99] Zhang YP Wang HY Zhang C Liu BP Peng ZL Li YY. Mifepristone attenuates depression-like changes induced by chronic central administration of interleukin-1beta in rats. Behav Brain Res. (2018) 347:436–45. doi: 10.1016/j.bbr.2018.03.033 29580890

[100] Wang N Ma H Li Z Gao Y Cao X Jiang Y. Chronic unpredictable stress exacerbates surgery-induced sickness behavior and neuroinflammatory responses *via* glucocorticoids secretion in adult rats. PloS One. (2017) 12:e0183077. doi: 10.1371/journal.pone.0183077 28806788 PMC5555668

[101] Shilpa BM Bhagya V Harish G Srinivas Bharath MM Shankaranarayana Rao BS. Environmental enrichment ameliorates chronic immobilisation stress-induced spatial learning deficits and restores the expression of BDNF, VEGF, GFAP and glucocorticoid receptors. Prog Neuropsychopharmacol Biol Psychiatry. (2017) 76:88–100. doi: 10.1016/j.pnpbp.2017.02.025 28288856

[102] Chen J Wang ZZ Zuo W Zhang S Chu SF Chen NH. Effects of chronic mild stress on behavioral and neurobiological parameters - Role of glucocorticoid. Horm Behav. (2016) 78:150–9. doi: 10.1016/j.yhbeh.2015.11.006 26592454

[103] Tsimpolis A Kokkali M Logothetis A Kalafatakis K Charalampopoulos I. Biphasic response of astrocytic brain-derived neurotrophic factor expression following corticosterone stimulation. Biomolecules. (2022) 12. doi: 10.3390/biom12091322 PMC9496348 36139161

[104] Numakawa T Adachi N Richards M Chiba S Kunugi H. Brain-derived neurotrophic factor and glucocorticoids: reciprocal influence on the central nervous system. Neuroscience. (2013) 239:157–72. doi: 10.1016/j.neuroscience.2012.09.073 23069755

[105] Popoli M Yan Z McEwen BS Sanacora G. The stressed synapse: the impact of stress and glucocorticoids on glutamate transmission. Nat Rev Neurosci. (2011) 13:22–37. doi: 10.1038/nrn3138 22127301 PMC3645314

[106] Numakawa T Odaka H Adachi N. Actions of brain-derived neurotrophic factor and glucocorticoid stress in neurogenesis. Int J Mol Sci. (2017) 18. doi: 10.3390/ijms18112312 PMC5713281 29099059

[107] Qian Q Liu Q Zhou D Pan H Liu Z He F. Brain-specific ablation of Efr3a promotes adult hippocampal neurogenesis *via* the brain-derived neurotrophic factor pathway. FASEB J. (2017) 31:2104–13. doi: 10.1096/fj.201601207R 28193719

[108] Walker AK Rivera PD Wang Q Chuang JC Tran S Osborne-Lawrence S. The P7C3 class of neuroprotective compounds exerts antidepressant efficacy in mice by increasing hippocampal neurogenesis. Mol Psychiatry. (2015) 20:500–8. doi: 10.1038/mp.2014.34 PMC4206684 24751964

[109] Sanacora G Treccani G Popoli M. Towards a glutamate hypothesis of depression: an emerging frontier of neuropsychopharmacology for mood disorders. Neuropharmacology. (2012) 62:63–77. doi: 10.1016/j.neuropharm.2011.07.036 21827775 PMC3205453

[110] Eisch AJ Petrik D. Depression and hippocampal neurogenesis: a road to remission? Science. (2012) 338:72–5. doi: 10.1126/science.1222941 PMC3756889 23042885

[111] Russell G Kalafatakis K Durant C Marchant N Thakrar J Thirard R. Ultradian hydrocortisone replacement alters neuronal processing, emotional ambiguity, affect and fatigue in adrenal insufficiency: The PULSES trial. J Intern Med. (2024) 295:51–67. doi: 10.1111/joim.13721 37857352 PMC10952319

[112] Frodl T Schule C Schmitt G Born C Baghai T Zill P. Association of the brain-derived neurotrophic factor Val66Met polymorphism with reduced hippocampal volumes in major depression. Arch Gen Psychiatry. (2007) 64:410–6. doi: 10.1001/archpsyc.64.4.410 17404118

